# BANK1 alters B cell responses and influences the interactions between B cells and induced T regulatory cells in mice with collagen-induced arthritis

**DOI:** 10.1186/s13075-017-1503-x

**Published:** 2018-01-25

**Authors:** Jie Yang, Jie Ren, Yiming Yang, Juan Sun, Xiaohui Zhou, Shucong Zheng, Dandan Xuan, Yu Xue, Huimin Fan, Jiong Zhang, Hejian Zou, Weiguo Wan, Ning Kong

**Affiliations:** 1 Department of Rheumatology, Huashan Hospital, Fudan University, No. 12 Wulumuqi Zhong Road, 200040 Shanghai, China; 2 Blood Engineering Lab, Shanghai Blood Center, Shanghai, China; 3 Research Center for Translational Medicine, Shanghai East Hospital, Tongji University School of Medicine, Shanghai, China

## Abstract

**Background:**

Functional variants of the B cell gene, B cell scaffold protein with ankyrin repeats 1 (BANK1) contribute to rheumatoid arthritis (RA) susceptibility, but their influences on B cell responses are unclear. Moreover, the function of induced T regulatory cells (iTregs) in the inflammatory milieu in a collagen-induced arthritis (CIA) model is unknown. This study was performed to investigate the roles of BANK1 in CIA and the interaction between B cells and iTregs.

**Methods:**

The changes in BANK1 mRNA and protein levels and their correlation with disease severity in CIA were determined. Next, the antigen-presenting function and autoantibody production in B cells were evaluated by co-culture with effector T cells and iTregs, respectively, both in vitro and in vivo. Then, the mechanisms underlying these interactions were studied by adding neutralizing antibodies or transwell inserts and by adoptive transfer to B-cell-depleted CIA mice.

**Results:**

The BANK1 level decreased in the peripheral blood, spleen and lymph nodes of CIA mice, particularly during the acute stage of arthritis, and exhibited negative correlation with disease severity and autoantibody production. B cell responses were enhanced by this decrease. B cells from CIA mice (CIA-B cells) promoted iTreg differentiation, proliferation and cytotoxic T lymphocyte-associated protein-4 (CTLA-4) expression. Meanwhile, BANK1 expression in CIA-B cells increased after co-culture with iTregs, limiting B cell responses. All these interactions depended on cell contact with CTLA-4-overexpressing iTregs but were independent of CTLA-4 cytokine.

**Conclusion:**

Decreased BANK1 expression promotes B cell responses, resulting in an increased antigen presentation ability and autoantibody production that subsequently influences the communication between B cells and iTregs through a cell-contact-dependent and CTLA-4- cytokine-independent mechanism in CIA mice.

## Background

Rheumatoid arthritis (RA) is an autoimmune disease characterized by progressive, destructive arthritis and ultimately causes joint dysfunction. Both T cells and B cells play an important role in RA pathogenesis [1–4]. Autoantibodies against rheumatoid factor (RF) and cyclic peptide containing citrulline (CCP) are the main adverse prognostic factors [5–7] of RA. Rituximab, a chimeric monoclonal IgG-1 antibody against the CD20 molecule expressed on B cells, is a well-known treatment for diseases with too many B cells, overactive B cells and dysfunctional B cells. This biological agent has been licensed for patients with RA who are refractory to first-line treatment [8, 9] and has confirmed the effects of B cells on this disease.

The B cell scaffold protein with ankyrin repeats 1 (BANK1) is expressed in B cells, but not T cells, and promotes tyrosine phosphorylation of the IP3 receptor to modulate B cell antigen receptor (BCR)-induced calcium mobilization [10]. BANK1 also weakens CD40-mediated Akt activation to prevent B cell hyperaction [11]. In some studies, functional variants of BANK1 are associated with autoimmune diseases such as systemic lupus erythematosus (SLE) and RA [12–15]. However, only a few studies have verified the roles of the BANK1 protein in autoimmune diseases and immune-associated diseases. Tineke Cantaert et al. explored the effects of alterations in BANK1 expression on humoral autoimmunity in arthritis but did not identify an important role [16]. Some scientists have noticed that higher BANK1 transcript levels help maintain stable immune tolerance in the absence of immunosuppression [17]. Based on these data, BANK1 may negatively affect immune-regulatory mechanisms in some diseases.

B cells interact with T cells through both BCRs and some molecules expressed on T cells that function as ligands [18]. This requires B cell antigen-presentation to T cells and serial interactions between receptor/ligand pairs belonging to CD28/B7 and cytokine superfamilies. They cooperate to induce optimum effector T cell activation and shut-down, to initiate regulatory T cell development and negative immune responses [19]. These interactions activate B cells to increase the expression of costimulatory factors and proliferation, subsequently promoting their differentiation into antibody-producing plasma cells [20]. B cells have also been shown to function as crucial antigen-presenting cells (APCs) that present certain antigens to initiate autoreactive T cells [21, 22] and are essential for self-reactive CD4^+^ T cell activation [23]. Meanwhile, self-reactive CD4^+^ T cells, which mainly react to B cells that express costimulatory molecules [24–26], are induced to differentiate into T helper cells (Th, which are also known as CD4^+^ T cells) such as Th17 and Th2 cells, which can produce considerably greater levels of pro-inflammatory factors and promote inflammatory disease progression. Any interruption of the interactions between B cells and T cells potentially contributes to the development of immune-deficient and autoimmune diseases [18].

Induced T regulatory cells (iTregs) exert excellent preventive and therapeutic effects on collagen-induced arthritis (CIA) and induce the production of additional suppressive cells after adoptive transfer in a CIA model in vivo [27], but the mechanism involved requires further exploration. In addition to T cells, regulatory T cells are also known to directly suppress B cells [28], and B cells are required for foxp3^+^ Treg expansion in the inflammatory milieu in B cell activation factor of the TNF family (BAFF) transgenic mice [29].

Although functional variants of BANK1, a negative regulator of B cells, are associated with RA [13], its physiological function in this disease is not clear. Based on the findings presented above, the present study was intended to evaluate BANK1 expression in peripheral B cells in the classic murine model of RA, the CIA mouse, its influence on changes in B cell phenotypes and correlation between BANK1 expression and the severity of arthritis. We hypothesized that these B cells from CIA mice (CIA-B cells) interact with self-reactive CD4^+^ T cells in a specific manner in CIA, which may enhance the inflammatory reactions in arthritis. We also assessed the effects of Tregs on these CIA-B cells and the contribution of the B cells as APCs to Tregs in the CIA inflammatory milieu.

## Methods

### Mice

All male DBA1/J mice used in this study were fed in the animal facility of the School of Pharmacy of Fudan University and the Shanghai Blood Center.

### Induction of CIA

Eight-week-old male mice were first immunized with subcutaneous injection of 75 μl of an emulsion containing a 1:1 ratio of bovine type II collagen (CII, Chondrex, Redmond, WA, USA) and Freund’s complete adjuvant (CFA, Difco, Detroit, MI, USA) on day 0 and were subsequently immunized with 50 μl of an emulsion containing a 1:1 ratio of CII and Freund’s incomplete adjuvant (IFA, Difco, Detroit, MI, USA) on day 21 after the first immunization. Clinical scores were recorded every other day to obtain clinical evidence of arthritis of the limb joints by a macroscopic examination from day 21 to day 49 after the first immunization. Limb joint arthritis was scored using an established scoring system [27] as follows: no detectable arthritis, 0; erythema and mild swelling, 1; mild erythema and mild swelling involving the entire paw, 2; severe swelling and redness from the ankle to digits, 3; and maximal swelling and redness or obvious joint destruction associated with visible joint deformity or ankylosis, 4. The clinical scores for each mouse are presented as the sum of the scores for the four limbs, and the maximum score for each mouse was 16. Two independent observers without knowledge of the experimental protocol performed the scoring. The clinical scores increased rapidly and constantly during the 49-day observation, and CIA developed in approximately 90% of mice.

### Enzyme-linked immunosorbent assay (ELISA) for anti-CII antibodies

Approximately 100 μl of peripheral blood obtained from the mouse inner canthus veniplex was collected every 7 days after immunization, clotted at room temperature for 1 h, and incubated in a 4 °C refrigerator overnight. The serum was then collected and stored at -80 °C. Titers of the anti-mouse CII antibody (anti-CII total IgG, anti-CII IgG2a and anti-CII IgG2b) in serum were measured using an ELISA kit (Chondrex, Inc. Redmond, WA, USA).

### Real-time polymerase chain reaction for the BANK1 mRNA

Total RNA was extracted from peripheral blood cells using an RNeasy Mini kit (TaKaRa Bio Inc., Japan). Complementary DNAs were generated with an Omniscript Reverse Transcription kit (TaKaRa Bio Inc., Japan), and BANK1 mRNA expression was quantified using the following primers (Invitrogen, Thermo Fisher Scientific Inc., MA, USA): forward: 5’-CAGACCTGCTGCATATTGCT-3’ and reverse: 5’-CTTGCTTGCTATTTCTGCCA-3’. PCR results were normalized to the expression of the housekeeping gene β-actin.

### Antibody staining and flow cytometry

Approximately 1 × 10^6^ cells collected from the spleen and lymph nodes of normal DBA1/J mice and arthritic mice (35 days after immunization) were incubated with anti-mouse CD80-FITC/CD86-FITC/MHC Class II (I-A/I-E)-PE and anti-mouse CD19-APC antibodies (BD Biosciences, San Jose, CA, USA), washed and detected by flow cytometry. Approximately 1 × 10^6^ cells collected from the spleen and lymph nodes of normal and immunized animals were incubated with an anti-mouse CD19-PE antibody (BD Biosciences, San Jose, CA, USA), fixed, permeabilized, and stained with anti-mouse BANK1-Alexa Fluor 647 or isotype control monoclonal antibodies (mAbs) (Santa Cruz Biotechnology, Inc. Santa Cruz, CA, USA and BD Biosciences, San Jose, CA, USA, respectively). The co-expression of BANK1 and CD19 was analyzed by flow cytometry. Naïve CD4^+^CD25^-^ cells isolated from spleen cells were used to generate CD4^+^Tregs and were labeled with an anti-mouse CD25-PE antibody (BD Biosciences, San Jose, CA, USA), fixed, permeabilized, stained with anti-mouse foxp3-APC or isotype control mAbs (eBioscience, San Diego, CA, USA) and analyzed by flow cytometry. Finally, iTregs labeled with carboxyfluorescein succinimidyl ester (CFSE) were stained with an anti-mouse cytotoxic T lymphocyte-associated protein-4 (CTLA-4)-PE antibody (BD Biosciences, San Jose, CA, USA) and evaluated by flow cytometry.

### Western blotting

Approximately 2 × 10^6^ cells from draining lymph nodes (inguinal and popliteus lymph nodes) from normal and CIA mice were lysed on ice with 30 μl of sodium dodecyl sulfate (SDS)-loading buffer. The resulting solution was collected, boiled at 100 °C for 5 min, and centrifuged for 1 min at 12,000 rpm (13,400 × g). The supernatant was used for western blot analysis of BANK1 levels. Equal amounts of protein from each sample were resolved on a 10% polyacrylamide gel by electrophoresis. Proteins were transferred to a polyvinylidene fluoride (PVDF) membrane. Membranes were blocked for 1 h at room temperature with 5% BSA in 1 × TBS with 0.1% Tween-20. Blots were incubated with an anti-BANK1 primary antibody (1:10 dilution; Santa Cruz Biotechnology) overnight at 4 °C, followed by 1 h incubation with the secondary antibody (horseradish peroxidase (HRP)-conjugated anti-rabbit IgG, 1:1000; Santa Cruz Biotechnology). The target proteins were visualized by enhanced chemiluminescence (ECL; Pierce, Thermo Fisher Scientific Inc., Rockford, IL, USA). Mouse brain extracts were used as standard controls.

### Immunohistochemical analysis

Draining lymph nodes (inguinal and popliteus lymph nodes) from naïve DBA1/J mice and CIA mice (14 and 35 days after immunization) were collected and fixed with formalin. Then, all fixed samples were embedded in paraffin, and 3-μm tissue sections were prepared. Sections were incubated overnight in an oven at 69 °C and dewaxed. Dewaxed sections were boiled in a preheated sodium citrate antigen repair solution for 20 min and kept warm for 10 min. Then, sections were washed three times with 1 × PBS, incubated with a 1:200 dilution of the anti-BANK1 antibody overnight at 4 °C, followed by an HRP-conjugated rabbit anti-mouse secondary antibody (Santa Cruz Biotechnology, Inc. Santa Cruz, CA, USA) for 30 min at 37 °C. Finally, sections were counterstained with hematoxylin, mounted and placed under coverslips.

### Cell preparation

#### Generation of CD4^+^ CD25^-^ T cells

CD4^+^ T cells were negatively selected from the spleen cells of DBA1/J mice using a CD4^+^ T Cell Isolation kit (Miltenyi Biotec Technology & Trading, Shanghai, China) and labeled with an anti-CD25-PE antibody (BD Biosciences, San Jose, CA, USA). Then, these cells were incubated with anti-PE-microbeads and isolated with a magnetic cylinder (Miltenyi Biotec Technology & Trading, Shanghai, China).

#### Generation of B cells

The spleen cells from naïve DBA1/J mice or CIA mice were collected on the 35th day after immunization and labeled with an anti-mouse CD19-PE antibody (BD Biosciences, San Jose, CA, USA), because the expression of CD80, CD86 and MHC II on B cells increased significantly at this time point. Then, these CD19^+^ cells were incubated with anti-PE-microbeads and isolated with a magnetic cylinder (Miltenyi Biotec Technology & Trading, Shanghai, China).

#### Generation of CD4^+^ iTreg cells

CD4^+^CD62L^+^CD25^-^ T cells were isolated from the spleen cells of naïve DBA1/J mice. Cells were cultured in RPMI 1640 medium supplemented with 10% fetal bovine serum (Invitrogen, Thermo Fisher Scientific Inc., MA, USA), 40 U/ml IL-2 (R&D Systems, Minneapolis, MN, USA), 2 ng/ml TGF-β (R&D Systems, Minneapolis, MN, USA) and CD3/CD28-pre-coated microbeads (Miltenyi Biotec Technology & Trading, Shanghai, China) at a 4:1 ratio for 4 days.

### Detection of pro-inflammatory factors using a cytometric bead array (CBA)

CD4^+^ T cells were cultured in RPMI 1640 medium supplemented with 100 U/ml IL-2 and 200 ng/ml type II collagen (CII) alone or with B cells from naïve DBA1/J mice treated in the presence or absence of anti-BANK1 antibody (N-B cells) or CIA-B cells at a 1:1 ratio for 3 days. Cells were stimulated with phorbol-12-myristate-13-acetate (PMA) (0.25 μg/ml) and ionomycin (0.25 μg/ml) for 5 h and brefeldin A (5 μg/ml) for 4 h at the end of the incubation period. Supernatants were harvested from each sample and IL-6, IL-17A and TNF-α were separately determined by CBA using a Mouse Soluble Protein Master Buffer Kit (BD Biosciences, San Jose, CA, USA).

### Proliferation assay

Set 1: CD4^+^ T cells were pre-labeled with CFSE (CellTrace™ CFSE Cell Proliferation Kit, Invitrogen, Germany) and co-cultured with CIA-B or N-B cells as APCs in the presence of 100 U/ml IL-2 and 200 ng/ml CII for 3 days. Then, the percentage of proliferative CD4^+^ T cells among total CFSE^+^ cells was detected by flow cytometry.

Set 2: iTregs were labeled with CFSE and co-cultured with CIA-B cells at 1:1 ratio in the presence of IL-2 (100 ng/ml) and CII (200 ng/ml) in the presence or absence of a purified anti-CTLA-4 neutralizing antibody (100 ng/ml; eBioscience, San Diego, CA, USA) or transwell inserts in 24-well plates. Approximately 1 × 10^6^ iTregs (the bottom layer) and 1 × 10^6^ B cells (the upper layer) were separated by the transwell inserts. Cells were harvested and analyzed by flow cytometry after 3 days.

### Statistical analysis

All data were analyzed using GraphPad Prism 5.0 software (GraphPad Software, San Diego, CA, USA). The unpaired *t* test was used to assess whether differences between two groups were statistically significant. One-way analysis of variance (ANOVA) was used to compare data between multiple groups. Correlation was analyzed using Spearman’s test. Linear regression analysis was performed when correlation was identified. An alpha value of *P* < 0.05 was considered significant.

## Results

### BANK1 expression in CIA

The BANK1 mRNA levels, the percentage of BANK1^+^CD19^+^ cells and the level of the BANK1 protein were evaluated with real-time PCR, flow cytometry, western blotting, and immunohistochemical analyses, respectively, to characterize BANK1 expression in CIA. Correlation between the BANK1 mRNA levels and arthritic scores and anti-CII antibodies was also analyzed.

**Fig. 1:**
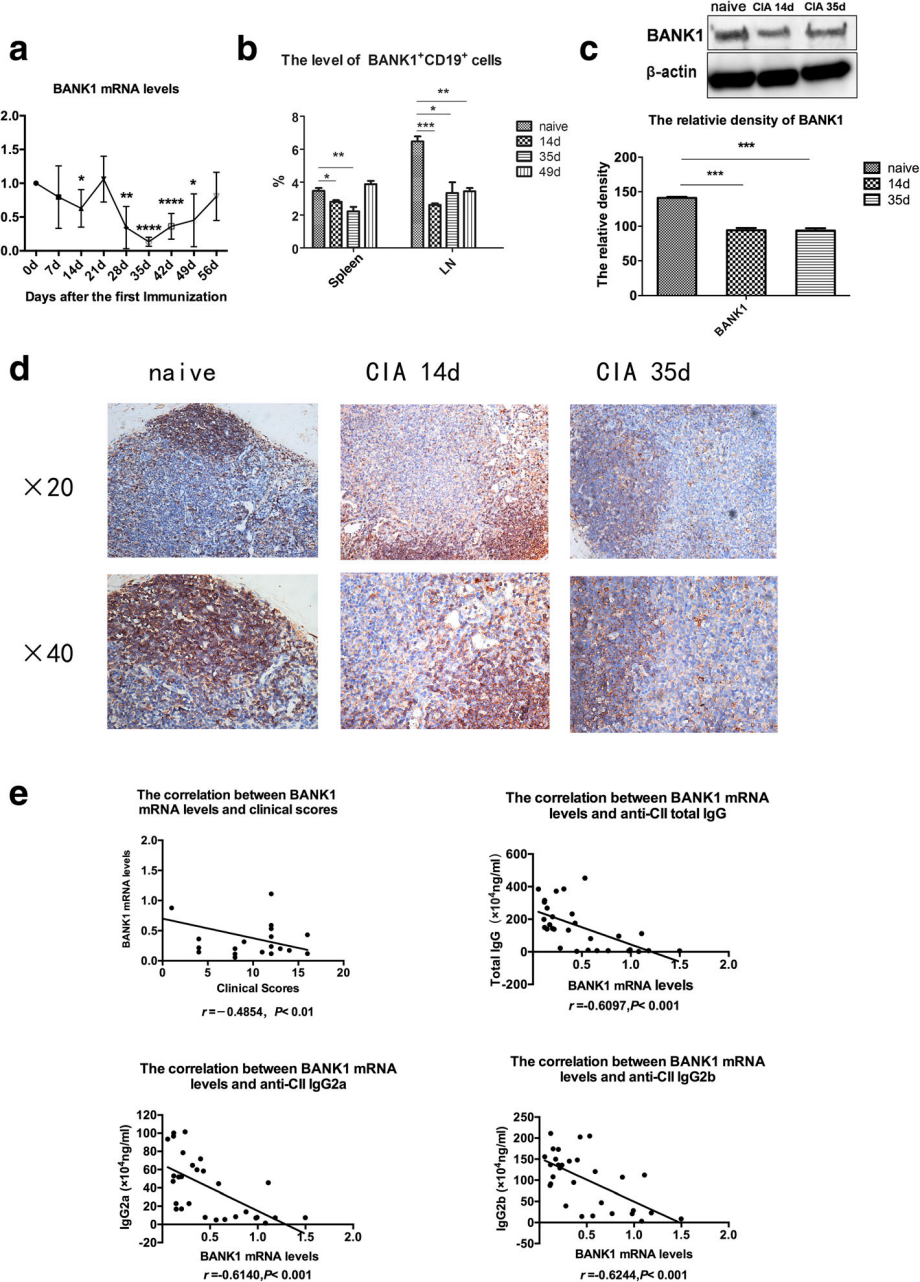
BANK1 expression in CIA. **a** BANK1 mRNA levels were detected by real-time PCR. **b** The percentage of BANK1^+^CD19^+^ cells was detected by flow cytometry. **c** The BANK1 protein level was evaluated by western blotting (WB), and the relative density is shown. **d** BANK1 expression in draining lymph nodes (LNs) in naive DBA1/J mice and collagen-induced arthritis (CIA) mice (14 and 35 days after immunization) was observed by immunohistochemical staining. BANK1 expression in lymphoid nodules in LNs from naïve DBA1/J mice and the swollen LNs from CIA mice 14 and 35 days (d) after immunization (original magnification × 20 and × 40). Representative data from one of three independent experiments are shown (**c**, **d**). **e** Correlation between BANK1 mRNA levels and clinical scores and also the titers of three anti-collagen type II (anti-CII) antibodies was analyzed. The results represent pooled data from three independent experiments. Bars show the means ± SEM. The post-hoc comparisons were done following one-way analysis of variance for repetitive testing. Correlation was analyzed using Spearman’s test. Linear regression analysis was performed when correlation was identified: *P < 0.05, **P < 0.01, ***P < 0.001, ****P < 0.0001

BANK1 mRNA levels initially decreased on the 14th day after immunization with bovine CII. The levels decreased again between the 28th and 49th days after immunization, particularly during the acute stage of arthritis (the 28th to 42nd days after immunization, Fig. 1a). Then, cells from the spleen and draining lymph nodes were labeled with anti-CD19-PE and anti-BANK1-IgG (H + L)-Alexa Fluor 647 antibodies as mentioned above and evaluated using flow cytometry. BANK1 was expressed at significantly lower levels in B cells on the 14th day (the beginning of the immune response to CII) and the 35th day (disease progression) after immunization (Fig. 1b), and BANK1 protein levels in draining lymph node cells (Fig. 1c) showed the same trend. BANK1 was expressed in naïve DBA1/J mice at much higher levels in lymphoid nodules, which primarily comprise B cells, than in CIA mice (14 days and 35 days after immunization), particularly in mice with acute exacerbation of arthritis (35 days after immunization) (Fig. 1d, original magnification × 20 and × 40). In addition, the BANK1 mRNA level was negatively correlated with arthritis clinical scores and the levels of the three main anti-CII antibodies (anti-CII total IgG, IgG2a and IgG2b antibodies, Fig. 1e).

### The role of CIA-B cells in effector T cell proliferation following CII-specific stimulation − one cause of CIA development and aggravation

**Fig. 2:**
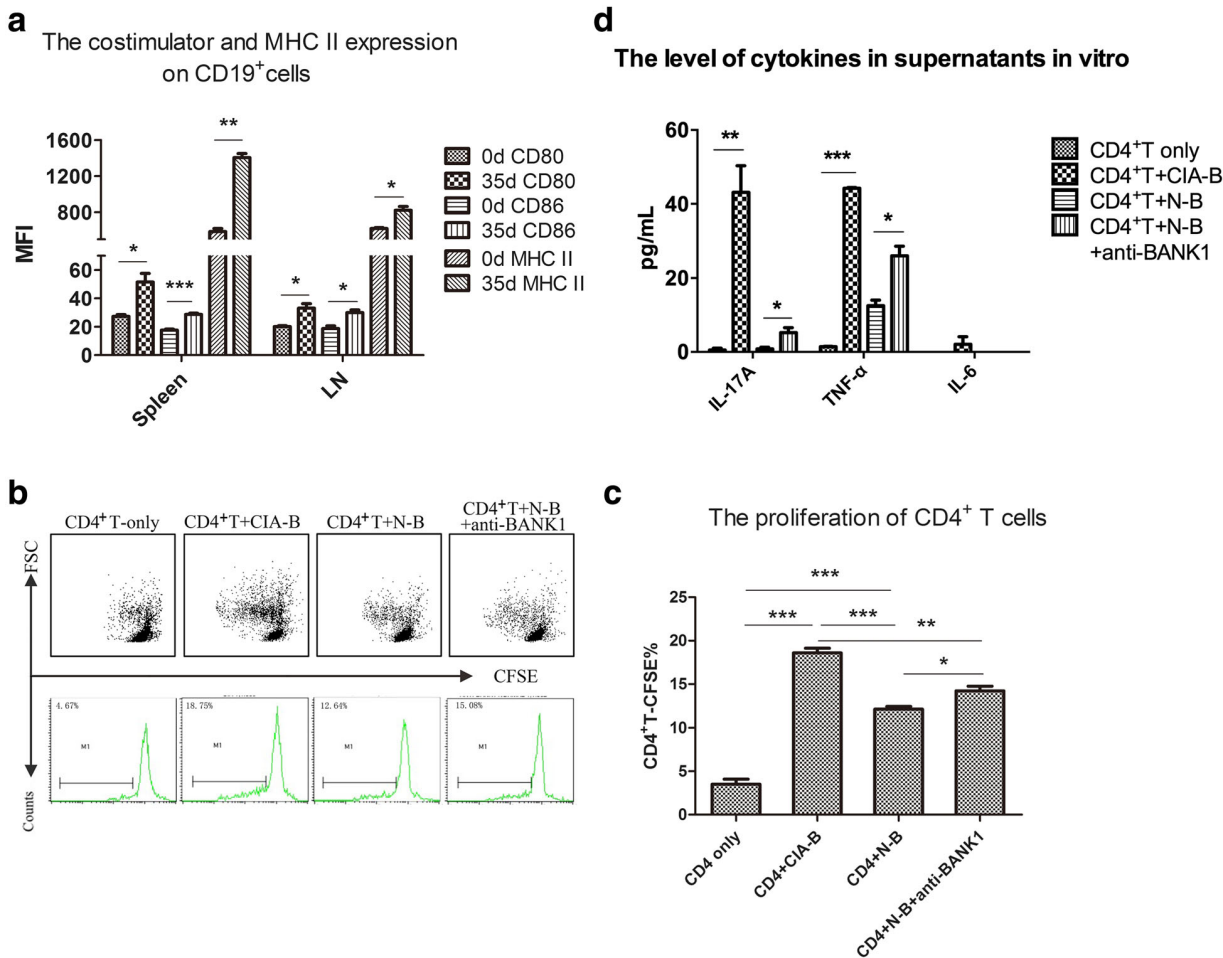
The role of B cells from mice with collagen-induced arthritis (CIA-B cells) in effector T cell proliferation following collagen type II (CII)-specific stimulation, which is one possible cause of CIA development and aggravation. **a** Costimulator and major histocompatibility II (MHC II) expression on CIA-B cells was analyzed by flow cytometry. The mean fluorescence intensity (MFI) of CD80, CD86 and MHC II on CD19^+^cells in spleen and lymph nodes (LNs) are shown. **b**, **c** The percentage of proliferative CD4^+^ carboxyfluorescein succinimidyl ester (CFSE)-labeled cells was detected by flow cytometry. **d** Supernatants were collected from each sample and the levels of pro-inflammatory factors (IL-17A, TNF-α and IL-6), which are closely related to arthritis progression, were evaluated using a cytometric bead array (CBA). At least five mice were included in each group. Bars show the means ± SEM of three independent experiments: *P < 0.05, **P < 0.01 and ***P < 0.001 for the comparisons of the indicated groups using the unpaired *t* test. *d* day, *FSC* forward scatter

Because the crucial function of activated B cells is to work as APCs that express high levels of costimulatory molecules (CD80 and CD86) and major histocompatibility complex II (MHC II), we noted that the expression of these molecules on B cells was significantly increased on the 35th day after immunization (Fig. 2a). Therefore, we used CII peptide-specific stimulation to confirm the functions of these cells as APCs that promote effector T cell proliferation.

Four groups were established: CD4^+^T-only, CD4^+^T + N-B, CD4^+^T + CIA-B and CD4^+^T + N-B + anti-BANK1 neutralizing antibody (100 ng/ml). CFSE-labeled CD4^+^ T cells were cultured with different types of B cells at a 1:1 ratio, 100 U/ml IL-2 and 200 ng/ml CII, for 3 days. The percentage of proliferative CFSE^+^ cells was detected by flow cytometry. CD4^+^ T cells that were co-cultured with CIA-B cells exhibited significantly greater proliferation rates than CD4^+^ T cells co-cultured with N-B cells (Fig. 2b, c; *P* < 0.001). In addition, the proliferation of CD4^+^ T cells promoted by N-B cells was partially reversed by the anti-BANK1 neutralizing antibody (Fig. 2b, c, *P* < 0.05). In addition, the supernatants were collected from each group, and CBAs were used to evaluate the levels of pro-inflammatory factors (IL-17A, TNF-α and IL-6), which are closely related to arthritis progression (Fig. 2d). The levels of IL-17A and TNF-α were dramatically increased in the CIA-B group compared with those in the N-B group, and the production of these two proteins was further increased when the neutralizing antibody (anti-BANK1 antibody, 100 ng/mL) was added to N-B cells (*P* < 0.05). However, IL-6 production was not altered.

**Fig. 3:**
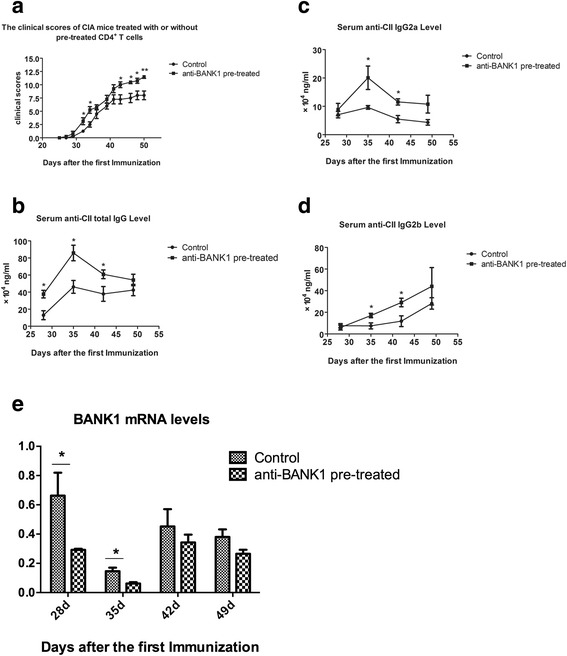
Confirmation of the effects of B cells from naïve DBA1/J mice (N-B; control) and anti-BANK1 antibody/N-B-treated CD4^+^ T cells (pre-treated CD4^+^ T cells) in vivo. **a** Clinical scores were recorded every other day after immunization. **b**-**d**. Anti-collagen type II (anti-CII) antibody levels in serum were detected using an ELISA. **e** The BANK1 mRNA levels in each group were detected. Bars show the means ± SEM of pooled data from three independent experiments. At least five mice were included in each group: *P < 0.05 and **P < 0.01 for comparison of the indicated groups using the unpaired *t* test. *CIA* collagen-induced arthritis, *d* days

CIA mice received an adoptive transfer of 5 × 10^6^ cells on the 25th day after immunization to confirm the effects of N-B (control) and anti-BANK1 antibody/N-B-treated CD4^+^ T cells (pre-treated CD4^+^ T cells) in vivo. The clinical scores were recorded every other day, and the anti-CII antibody titer and BANK1 mRNA levels were determined as described above. Mice that received pre-treated CD4^+^ T cells had earlier disease onset, greater disease severity and a higher deformity rate than control mice (Fig. 3a) in addition to lower BANK1 mRNA levels and higher anti-CII antibody titer (Fig. 3b-e).

### The communication between iTregs and CIA-B cells in vitro and in vivo

According to previous studies, iTregs exert excellent preventive and therapeutic effects on CIA [15, 18], including reducing the levels of pro-inflammatory factors and the anti-CII antibody. In addition, iTregs induce the generation of a greater number of suppressive cells in the inflammatory milieu in arthritic mice, but the underlying mechanism is still unclear. Based on previous findings and the aforementioned changes in CIA-B cells, we hypothesized that there is a crucial interaction between iTregs and CIA-B cells.

#### Effects of iTregs on CIA-B cells in vitro

**Fig 4:**
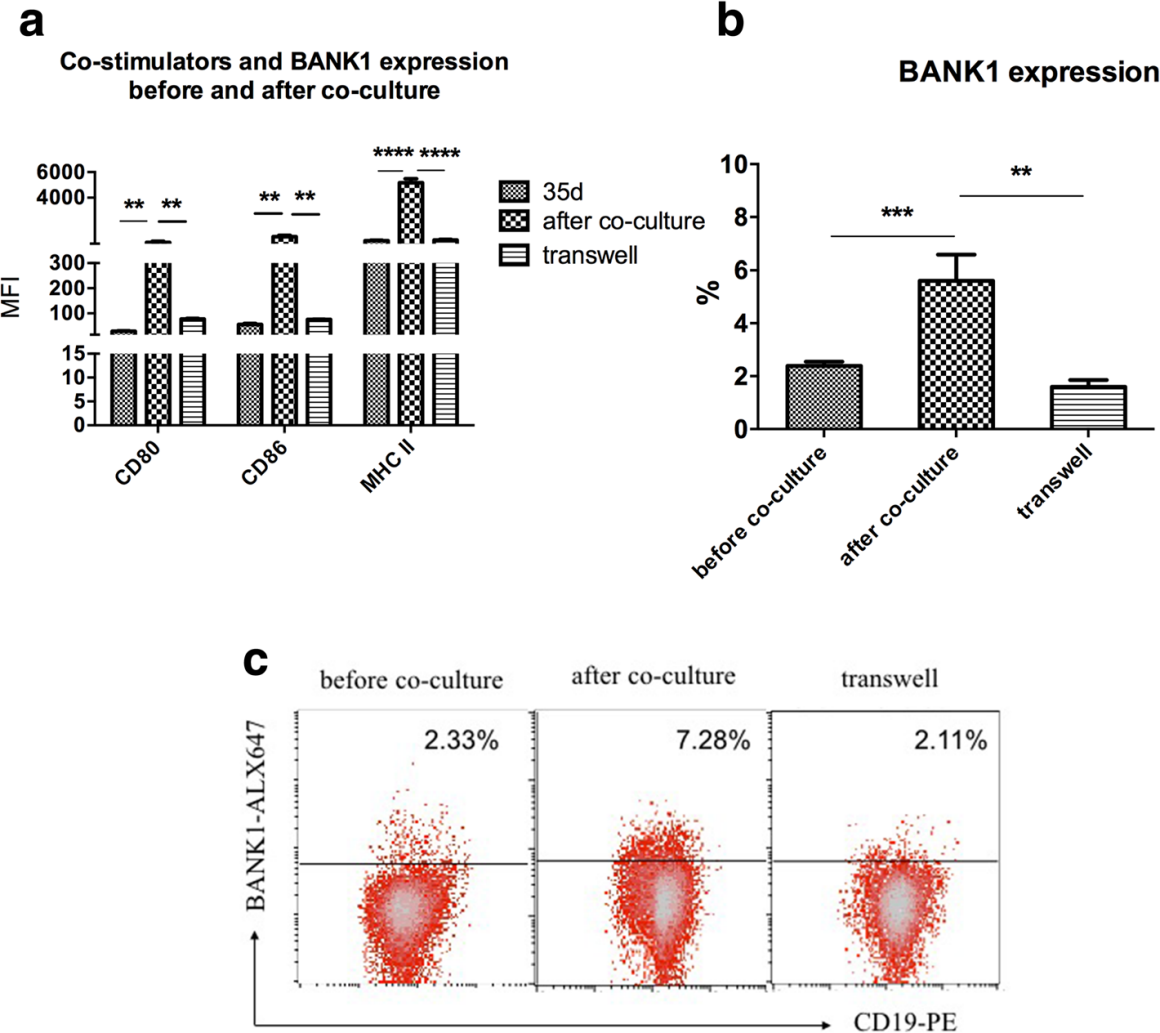
Effects of induced T regulatory cells (iTregs) on B cells from collagen-induced arthritis (CIA) mice (CIA-B cells) in vitro. **a** The mean fluorescence intensity (MFI) of costimulators and major histocompatibility complex II (MHC II) and the percentage of BANK1^+^CD19^+^ were detected by flow cytometry. **b**, **c** The percentages of BANK1^+^CD19^+^ cells in different groups were detected by flow cytometry. Bars show the means ± SEM of pooled data from six independent experiments: **P < 0.01, ***P < 0.001 and ****P < 0.0001 for comparison of the indicated groups using the unpaired *t* test. *d* days

Tregs were induced using a standard method, and B cells were isolated from spleen cells of CIA mice on the 35th day after immunization, when CD80, CD86 and MHC II expression on B cells were all significantly increased. Three groups were investigated: CIA-B, iTregs + CIA-B and iTregs + CIA-B + transwell. iTregs were labeled with CFSE and co-cultured with CIA-B cells at a 1:1 ratio in the presence of 100 U/ml IL-2 and 200 ng/ml CII for 3 days. Strikingly, the expression of these two costimulatory molecules and MHC II increased dramatically after co-culture with iTregs and returned to a basal level when the co-cultured cells were separated by transwell inserts (Fig. 4a). The same result was obtained when BANK1 expression was evaluated by flow cytometry (Fig. 4b, c). These effects were not reversed by the addition of the anti-CTLA-4 neutralizing antibody (data not shown).

#### CIA-B cells promote iTreg proliferation and differentiation in vitro and in vivo

Considering the impressive increase in the expression of costimulators and MHC II on CIA-B cells induced by iTregs, which enhances the antigen-presenting function of CIA-B cells, we evaluated whether this change promoted iTreg differentiation and proliferation.

**Fig. 5:**
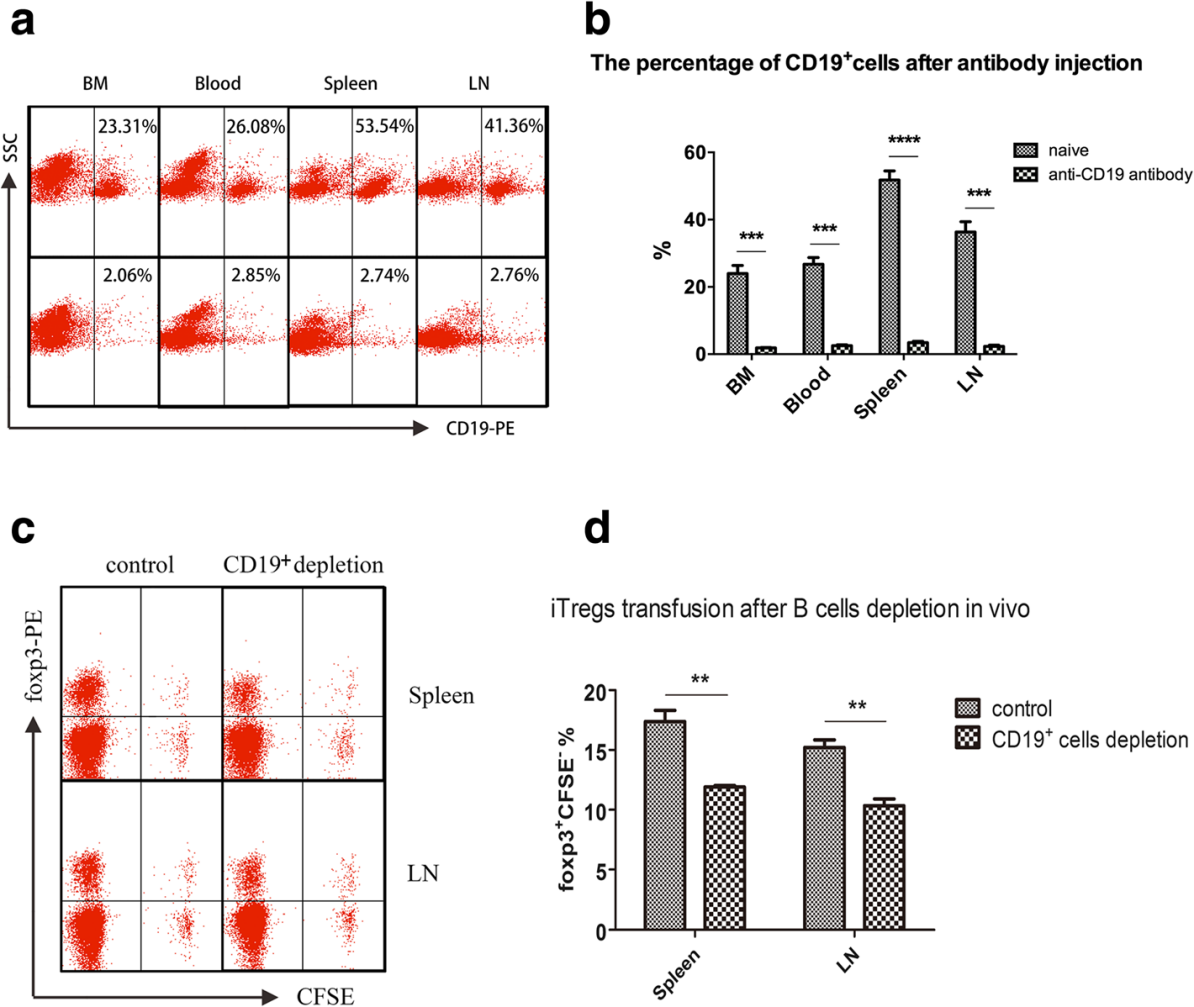
Contribution of B cells from collagen-induced arthritis (CIA) mice (CIA-B cells) to induced T regulatory cells (iTregs) in vivo. **a**, **b** The percentages of CD19^+^ cells in the bone marrow (BM), peripheral blood, spleen and draining lymph nodes (LNs) were detected by flow cytometry 7 days after the antibody injection. **c**, **d** The percentage of foxp3^+^ carboxyfluorescein succinimidyl ester (CFSE)^-^ cells in the spleen and draining LNs of CIA mice with or without B cell depletion was detected by flow cytometry 3 days after the adoptive transfer of iTregs. The results represent two independent experiments with five mice per group. Bars show the means ± SEM: **P < 0.01, ***P < 0.001 and ****P < 0.0001 for the comparisons of the indicated groups using the unpaired *t* test

CIA mice were injected with a purified anti-CD19 monoclonal antibody or purified IgG2a, κ isotype antibody (150 μg/mouse) on the 35th day after immunization to determine how CIA-B cells influence Tregs. B cells were almost completely depleted (Fig. 5a, b) from the bone marrow (BM), peripheral blood, spleen and draining lymph nodes 7 days after the anti-CD19 antibody injection. CFSE-labeled iTregs were adoptively transferred to the CIA mice (5 × 10^6^ cells/mouse) on the same day. The mice were killked 3 days after transfer, and then spleen and draining lymph node cells were stained with an anti-foxp3-PE antibody. An obviously higher percentage of foxp3^+^CFSE^-^ cells were detected in B cell-depleted CIA mice than in control mice (Fig. 5c, d, *P* < 0.01).

**Fig. 6:**
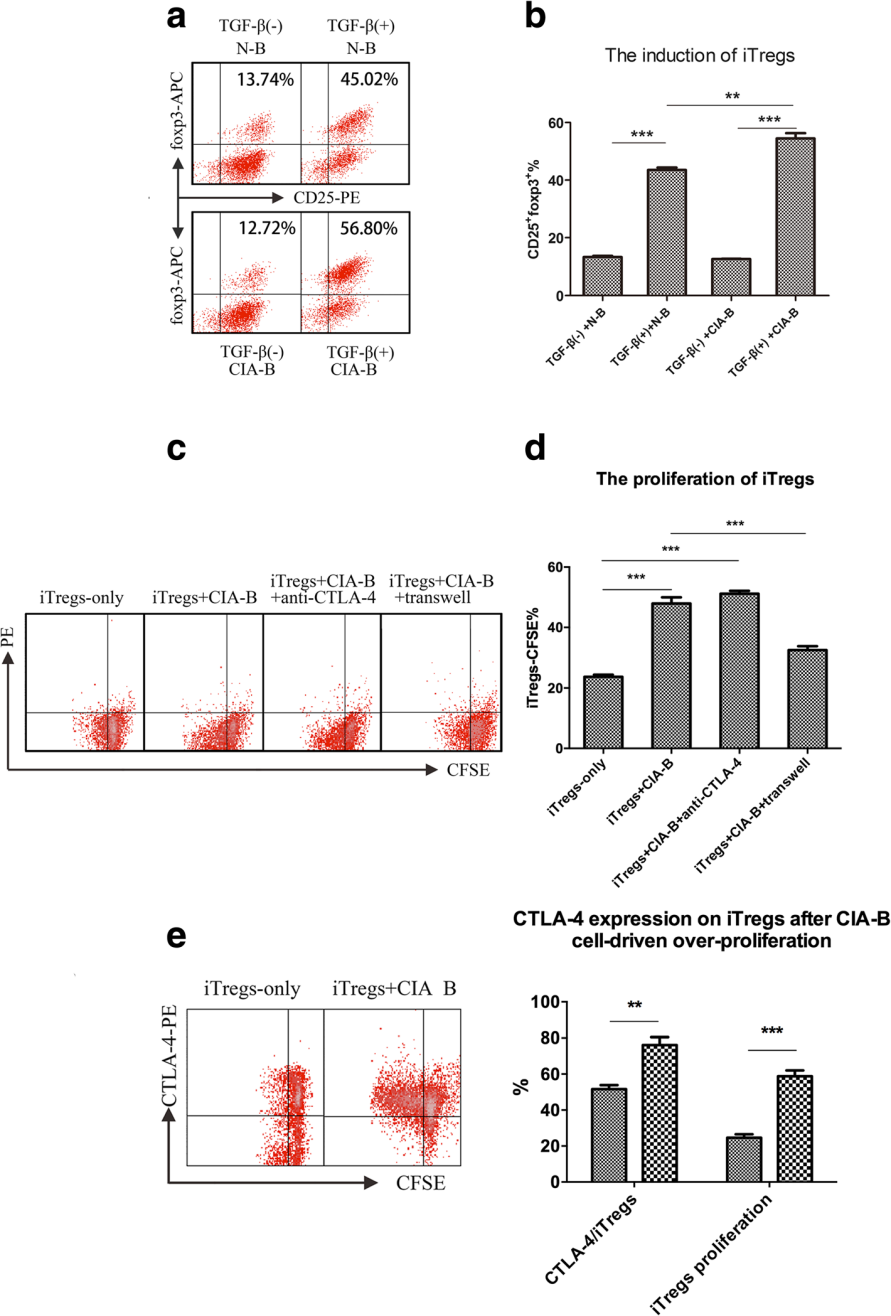
The mechanism underlying the effects of B cells from collagen-induced arthritis (CIA) mice (CIA-B cells) on induced T regulatory cells (iTregs). **a**, **b**. CIA-B cells influenced Treg induction in vitro. The percentage of CD4^+^CD25^+^foxp3^+^ cells was detected by flow cytometry after 3 days of co-culture with CIA-B or N-B cells in the presence or absence of transforming growth factor (TGF)-β. **c**, **d** CIA-B cells influenced Treg proliferation in vitro. The percentage of carboxyfluorescein succinimidyl ester (CFSE)-labeled iTregs was analyzed by flow cytometry after 3 days of co-culture with CIA-B cells, and some of the medium was treated with the anti-cytotoxic T-lymphocyte-associated protein-4 (anti-CTLA-4) neutralizing antibody or the cells were separated by transwell inserts. **e** CTLA-4 expression on iTregs after CIA-B cell-driven over-proliferation was determined by flow cytometry. The results represent pooled data from three independent experiments. Bars show the means ± SEM: **P < 0.01 and ***P < 0.001 for comparison of the indicated groups using the unpaired *t* test. *APC* antigen-presenting cells, *N-B* B cells from naïve DBA1/J mice

We performed two in vitro experiments to explore the mechanisms by which CIA-B cells induce Tregs. In the first experiment, we evaluated the effects of CIA-B cells on Treg induction. CD4^+^CD62L^-^ cells (naïve CD4^+^ T cells), N-B cells and CIA-B cells were isolated as described in “Methods”, and naïve CD4^+^CD25^-^ T cells were cultured with CIA-B or N-B cells at a 1:1 ratio along with 100 U/ml IL-2 in the presence or absence of 5 ng/ml TGF-β for 3 days. CIA-B cells induced the differentiation of a greater number of CD4^+^CD25^+^foxp3^+^ cells than N-B cells (*P* < 0.01, Fig. 6a, b). In the second experiment, we evaluated the effects of CIA-B cells on iTreg proliferation. CFSE-labeled iTregs were co-cultured with CIA-B cells in the presence or absence of the purified anti-CTLA-4 neutralizing antibody (100 ng/ml) or with transwell inserts. A greater number of iTregs co-cultured with CIA-B cells proliferated than iTregs cultured with N-B cells, proliferation was clearly reduced in the group separated by transwell inserts, and no change was noted when anti-CTLA-4 neutralizing antibody was added (Fig. 6c, d). Intriguingly, CTLA-4 expression was clearly upregulated from approximately 40 to 88% on iTregs and iTregs proliferation increased from 26 to 57% after co-culture with CIA-B cells (Fig. 6e).

## Discussion

BANK1 is a negative regulatory protein that prevents B cell over-activation and regulates B cell responses [11]. BANK1 single nucleotide polymorphisms contribute to autoimmune disease susceptibility in diseases such as systemic lupus erythematosus (SLE) and RA [12–15], both of which have B cells involved in their pathogenesis and progress [1, 2, 30, 31]. In the only relative research on RA, the scientists did not identify a positive impact of BANK1 protein on this chronic arthritis [16]. However, the other work on kidney transplantation that confirmed the contribution of B cells to immune tolerance attracted our attention [17]. This group first reported BANK1 as one of the key leader genes upregulated in patients with “true” tolerance to allografts without taking immunosuppressant drugs [32]. Then, they identified greater BANK1 molecule expression in CD19^+^ B cells and demonstrated its negative regulation of B cell responses [17]. Although post-kidney transplantation and RA are two different conditions, B cells are involved in the pathogenesis of each of them [1, 2, 17]. In this study, BANK1 mRNA and protein levels were clearly lower in the acute stage of CIA and were negatively correlated with clinical scores and anti-CII antibodies (Fig. 1). These findings suggested that BANK1 plays a regulatory role in CIA. Then, we speculated on the changes in B cells, considering the physiological function of BANK1 protein.

Notably, the expression of costimulatory factors (CD80 and CD86) and the MHC-II molecule was significantly increased on B cells from the spleen and lymph nodes in mice with established arthritis on the 35th day after immunization (Fig. 2a). B cells, which secrete antibodies in the humoral immune system and present antigens as professional APCs, play a key role in maintaining the human adaptive immune system [20, 21]. The activity of autoimmune diseases, including RA and SLE, is correlated with B cell activity [33]. B cell activation occurs in the secondary lymphoid organs (SLOs), such as the spleen and lymph nodes. As one kind of professional APC, B cells are characterized by the expression of costimulatory molecules and MHC class II [34], which are responsible for their antigen-presenting capacity. The ability of B cells to act as APCs was enhanced in CIA mice. A B cell recognizes an antigen that is then phagocytosed, and the B cell presents peptides using MHC class II molecules to a T cell. Activated B cells express higher levels of costimulatory molecules [35] required for T cell activation, including CD80 (B7-1) and CD86 (B7-2). These two B7 molecules can interact with CD28 on the surface of a CD4^+^ T cell [19]. We first detected the effects of BANK1-regulated CIA-B cells (B cells from CIA mice with established arthritis) on CD4^+^ T cells isolated from DBA1/J mice in the presence of the specific CII antigen, which stimulates an antigen-specific reaction, to determine the function of these cells. The CIA-B cells expressing higher levels of costimulatory factors increased CD4^+^ T cell proliferation and pro-inflammatory factor production (IL-17A and TNF-α, which are involved in CIA) in vitro and induced more pro-inflammatory effects in vivo than N-B cells (Figs. 2b, d and Fig. 3). When the anti-BANK1 neutralizing antibody was applied, the stimulatory capacity of normal B cells was enhanced (Fig. 2b, d). Thus, we concluded that the lack of BANK1 contributes to strengthening the B cell antigen-presenting function to Th cells in CIA mice.

T cells are very important for B cell activation and proliferation [36]. With the exception of CD4^+^T cells, Tregs, the suppressive T cell subset, interact with B cells as well. Notably, iTregs directly suppress B cells by regulating the CD28/CD80/86/CTLA-4 balance [19]. B7 proteins CD80 and CD86 expressed on B cells provide critical costimulatory or inhibitory input to T cells via their T cell-expressed receptors: CD28 and CTLA-4. CTLA-4, also known as CD152, is a protein receptor that functions as an immune checkpoint to downregulate immune responses. The combination of B7/CD28 signaling is required for CD4^+^T cell proliferation and function, whereas CTLA-4/CD28 signaling is essential for the negative regulation of adaptive immune responses by inhibiting effector T cell activation and Treg cell development and suppressive functions [37, 38]. The iTregs that were induced from CD4^+^ CD62L^+^CD25^-^ T cells by IL-2 and TGF-β possess a greater suppressive function in controlling CIA and induce the production of a greater number of foxp3^+^ suppressive cells in the inflammatory milieu in CIA mice than natural Tregs [27, 39]. However, the underlying mechanisms are not very clear. We tried to understand the interaction between iTregs and CIA-B cells both in vitro and in vivo. Strikingly, the expression of costimulatory molecules and the MHC II molecule on CIA-B cells was dramatically increased after co-culture with iTregs and decreased to nearly baseline levels when a transwell was inserted in vitro (Fig. 4a). The same trend was noted for BANK1 expression in B cells (Fig. 4b-c). Thus, iTregs might promote the CIA-B cell antigen-presenting capacity through a cell-cell contact-dependent mechanism and simultaneously support BANK1 expression to prevent B cell hyperactivation.

According to Walters S et al, BAFF-activated B cells directly suppress the effector functions of T cells by promoting the expansion of foxp3^+^ Tregs [29]. Because both the BAFF-Tg mice and CIA mice have an inflammatory internal environment, we attempted to determine whether the CIA-B cells displaying upregulation of antigen-presenting components had a more substantial contribution to iTreg activation and proliferation, and, conversely, how the CIA-B cells affected iTregs in this process. A B-cell-depleted murine model was generated [40] (Fig. 5a, b), and a lower percentage of foxp3^+^CFSE^-^ cells was detected in B-cell-depleted CIA mice than in controls (Fig. 5c-d). These foxp3^+^CFSE^-^ cells may be derived from two sources. One source was the CFSE-labeled iTregs that were adoptively transferred to CIA mice and lost their fluorescence when they proliferated in vivo; the other source was foxp3^+^ cells that were induced in vivo after the adoptive transfer. The in vitro experiments verified that CIA-B cells could induce the production of a greater number of CD25^+^foxp3^+^ cells than N-B cells under the same culture conditions and directly promoted iTreg proliferation (Fig. 6a-d). Interestingly, iTregs expressed CTLA-4 after co-culture with CIA-B cells, and the effect on iTreg proliferation was not counteracted by an anti-CTLA-4 neutralizing antibody but was counteracted by the transwell insert (Fig. 6e). CTLA-4 is constitutively expressed in regulatory T cells [41], and it acts as an “off” switch when bound to CD80 or CD86 on the surface of APCs [42]. Therefore, iTregs upregulated CTLA-4 expression after activation by CIA-B cells and negatively regulated the CD80/CD86/CTLA-4 balance through a cell-cell contact-dependent mechanism, but not a CTLA-4 cytokine-dependent mechanism.

## Conclusions

In this study, B cell responses were enhanced due to decreased BANK1 levels in CIA mice. We obtained improved understanding of the interactions between B cells, iTregs and T cells in this murine model. Lack of the BANK1 protein strengthened the B cell antigen-presenting ability, which contributed to iTreg proliferation, induction from CD4^+^ T cells and suppressive functions. Promotion of iTreg proliferation increased CTLA-4 expression, which subsequently bound to B7 proteins (also known as CD80 and CD86, the costimulators) with significantly higher affinity than CD28 (expressed on CD4^+^T cells). This interaction would enhance the suppressive function of iTregs. We confirmed that this interaction depended on cell-cell interactions, but not the CTLA-4 cytokine. Simultaneously, iTregs upregulated BANK1 expression to prevent B cell over-activation. We are the first to observe the role of BANK1 in CIA mice, thus improving our understanding of the interactions between B cells, iTregs and T cells in this inflammatory milieu. This information will be useful for enhancing our knowledge of the pathogenesis of RA/CIA and iTreg therapeutic capacity in CIA, and of the possibilities for immunotherapy for RA.

## Abbreviations

anti-CCP: Anti-cyclic peptide containing citrulline
APCs: Antigen-presenting cells
BAFF: B cell activation factor of the TNF family
BANK1: B cell scaffold protein with ankyrin repeats 1
BCRs: B cell antigen receptors
BSA: Bovine serum albumin
CII: Type II collagen
CBA: Cytometric bead array
CFA: Freund’s complete adjuvant
CFSE: Carboxyfluorescein succinimidyl ester
CIA: Collagen-induced arthritis
CIA-B: B cells from collagen-induced arthritis DBA1/J mice
CTLA-4: Cytotoxic T-lymphocyte-associated protein-4
ELISA: Enzyme-linked immunosorbent assay
FSC: Forward scatter
HRP: Horseradish peroxidase
IFA: Freund’s incomplete adjuvant
IL: Interleukin
iTregs: Induced T regulatory cells
mAbs: Monoclonal antibodies
MFI: Mean fluorescence intensity
MHC II: Major histocompatibility complex II
N-B: B cells from naïve DBA1/J mice
PBS: Phosphate-buffered saline
PMA: Phorbol 12-myristate 13-acetate
RA: Rheumatoid arthritis
RF: Rheumatoid factor
TGF: Transforming growth factor
Th cells: T helper cells
TNF: Tumor necrosis factor
